# Antibiotic Resistance and Extended-Spectrum β-Lactamases in Isolated Bacteria from Seawater of Algiers Beaches (Algeria)

**DOI:** 10.1264/jsme2.ME11266

**Published:** 2011-11-16

**Authors:** Souhila Alouache, Mohamed Kada, Yamina Messai, Vanesa Estepa, Carmen Torres, Rabah Bakour

**Affiliations:** 1 Laboratoire de Biologie Cellulaire et Moléculaire, Faculté des Sciences Biologiques, Université des Sciences et de la Technologie Houari-Boumédiène, B.P. 32 El-Alia, Bab-Ezzouar 16111, Alger, Algeria; 2 Ecole Nationale Supérieure des Sciences de la Mer et de l’Aménagement du Littoral, BP19 Campus Universitaire de Dély-Ibrahim, Alger, Algeria; 3 Área de Bioquímica y Biología Molecular, Universidad de La Rioja, 26006 Logrono, Spain

**Keywords:** antibiotic resistance, bacteria, seawater, beach

## Abstract

The aim of the study was to evaluate bacterial antibiotic resistance in seawater from four beaches in Algiers. The most significant resistance rates were observed for amoxicillin and ticarcillin, whereas they were relatively low for ceftazidime, cefotaxime and imipenem. According to sampling sites, the highest resistance rates were recorded for 2 sites subjected to chemical and microbiological inputs (amoxicillin, 43% and 52%; ticarcillin, 19.6% and 47.7%), and for 2 sites relatively preserved from anthropogenic influence, resistance rates were lowest (amoxicillin, 1.5% and 16%; ticarcillin, 0.8% and 2.6%). Thirty-four bacteria resistant to imipenem (*n*=14) or cefotaxime (*n*=20) were identified as *Pseudomonas aeruginosa* (*n*=15), *Pseudomonas fluorescens*(7), *Stenotrophomonas maltophilia*(4), *Burkholderia cepacia*(2), *Bordetella* sp. (1), *Pantoea* sp. (1), *Acinetobacter baumannii*(1), *Chryseomonas luteola*(1), *Ochrobactrum anthropi*(1) and *Escherichia coli*(1). Screening for extended spectrum β-lactamase showed the presence of CTX-M-15 β-lactamase in the *E. coli* isolate, and the encoding gene was transferable in association with the IncI1 plasmid of about 50 kbp. Insertion sequence IS*Ecp1B* was located upstream of the CTX-M-15 gene. This work showed a significant level of resistance to antibiotics, mainly among environmental saprophytic bacteria. Transmissible CTX-M-15 was detected in *E. coli*; this may mean that contamination of the environment by resistant bacteria may cause the spread of resistance genes.

Antibiotic-resistant bacteria are present in significant frequencies in various environments (22, 47); therefore, antibiotic resistance has become an ecological reality. This is a result of resistance genes and antibiotics or other antimicrobials (antiseptics, disinfectants, heavy metals etc.) released in natural ecosystems in concentrations leading to the selective survival of resistant bacteria (22, 23, 27). Moreover, these antimicrobials promote the dissemination of resistance genes by horizontal gene exchange mediated by plasmids and recombination events such as transposition and integration (9, 40).

Gram-negative bacteria in the environment, such as *Enterobacteriaceae*, *Vibrionaceae Pseudomonadaceae*, *Shewanellaceae*, *Burkholderiaceae*, *Xanthomonadaceae* and *Moraxellaceae*, were found to be resistant to major classes of antibiotics such as β-lactams and quinolones and their involvement in infectious diseases is a cause of treatment failure (4, 24). This undesirable evolution of bacteria exposes humans to health risks; indeed, aquatic environments can constitute a reservoir or a return path of microorganisms that can reach humans through the food chain (22, 26, 47) or recreational activities (31, 35). This is an emerging public health concern which requires the evaluation of antibiotic resistance in human-related aquatic environments.

The beaches of marine coasts are dynamic environments subject to natural changes in terms of physico-chemicals and nutrients and to strong anthropogenic pressure from various activities, and bacteria inhabiting these ecosystems are well adapted to these versatile conditions. Studies on water coastal quality have often limited their focus to the load and diversity of microbial populations, and antibiotic resistance evaluations have mainly been conducted on freshwater rivers, lakes, estuaries, drinking water and wastewater (1, 11, 13, 21, 44), while there have been few studies of marine waters. The aim of this study was to determine the prevalence of antibiotic resistance, particularly to β-lactams, in isolated bacteria from seawater at four beaches in Algiers with different levels of anthropogenic inputs.

## Materials and Methods

### Presentation of sampling sites

The samples were taken during March 2009 at four different sites. The mouth of Réghaia’s lake and the beach of Ain chrob are located in eastern Algiers; the first is the recipient of numerous streams and discharges from a sewage treatment plant in Réghaia city and the second is adjacent to a populated area and an offshore shellfish farming site. The beaches of Ain Tagourait and Anse de Kouâli are located in the bay of Bou-Ismail city in western Algiers; the first in an agricultural and shellfish farming area and the second is part of a nature reserve.

### Sample collection and water analysis

Water samples were taken from beaches 1 m from the waterline and at a depth of about 20 cm using 500 mL sterile glass bottles, and they were transported in cold bags at 4°C to the laboratory within 6 h. Salinity, pH, dissolved oxygen and temperature were measured *in situ* at the sampling sites, and suspended matter and biological oxygen demand (BOD) were determined in the laboratory (41).

The water microbiological quality was assessed by estimating the total flora on Mueller Hinton medium and total and thermotolerant coliforms on Tergitol medium, using the membrane filtration technique (41).

### Antibiotic resistance analysis

The prevalence of resistance to amoxicillin (AMX), ticarcillin (TIC), cefotaxime (CTX), ceftazidime (CAZ), cefoxitin (FOX) and imipenem (IMP) was determined using bacteria contained in 100 mL water that was first filtered onto the surface of 0.45 μm-pore membranes. Filters were incubated on Mueller Hinton agar medium with or without antibiotics at critical concentrations of 16 μg mL^−1^ for AMX, 64 μg mL^−1^ for TIC, 2 μg mL^−1^ for CTX, 8 μg mL^−1^ for CAZ and IMP, and 32 μg mL^−1^ for FOX (7). The prevalence of resistant bacteria was estimated by comparing the number of bacteria growing on medium with antibiotics with the number of bacteria growing on medium without antibiotics (13).

Gram-negative bacteria resistant to CTX or IMP, antibiotic markers of the production of extended spectrum β-lactamase (ESBL) or carbapenemases, were identified by API20E and API20NE systems (Biomerieux, Marcy l’Etoile, France) and their complete antibiotic resistance profile was determined. An antibiogram was performed by the disk-diffusion method on Mueller Hinton agar plates and interpreted according to the guidelines of the Antibiogram Committee of the French Society for Microbiology (7). The following antibiotic disks (Bio-Rad, Hercules, CA, USA) were used (μg or International Unit “IU”/disk): AMX, 25 μg; TIC, 75 μg; piperacillin (PIP) 75 μg; aztreonam (ATM) 30 μg; cefsulodine (CFS) 30 μg; CTX, 30 μg; ceftriaxone (CRO) 30 μg; CAZ, 30 μg; FOX, 30 μg; cefepime (FEP) 30 μg; IMP, 10 μg; piperacillin/tazobactam (PTZ) 75/10 μg; amoxicillin/clavulanic acid (AMC) 20/10 μg; ticarcillin/clavulanic acid (TCC) 75/10 μg; kanamycin (K) 30 IU; gentamicin (GM) 15 μg; sulfonamides (SSS) 200 μg; trimethoprim (TMP) 5 μg; trimethoprim/sulfamethoxazole (SXT) 1.25/23.75 μg; tetracyclines (TE) 30 IU; nalidixic acid (NA) 30 μg; ciprofloxacin (CIP) 5 μg; chloramphenicol (C) 30 μg and rifampicin (RA) 30 μg. *E. coli* ATCC 25922 was used as a control strain for antimicrobial susceptibility testing.

### Screening and identification of extended spectrum β-lactamases (ESBLs)

ESBL production was detected using the double-disk synergy test (DDST) as a standard disk-diffusion assay on Mueller-Hinton agar. Disks containing ATM, CAZ, CRO and CTX were placed at a distance of 30 mm (center to center) around a disk containing AMC. A synergistic effect between clavulanic acid and test antibiotics resulting in an increase of the inhibition zone toward the AMC acid disk is indicative of ESBL production (18).

Isolates positive for DDST were subjected to DNA extraction by the boiling method and screened for *bla*_TEM_ and *bla*_CTX-M_ genes by PCR using universal primers of CTX-M and TEM and specific primers for diverse CTX-M groups (CTX-M-1, CTX-M-2, CTX-M-9 and CTX-M-25 groups) as previously described (20). Cycling conditions were as follows: initial denaturation at 94°C for 5 min followed by 30 cycles of denaturation at 94°C for 40 s, annealing at 55°C for 30 s, and elongation at 72°C for 40 s. The final elongation step was extended to 10 min at 72°C. The PCR products were separated on 1.5% agarose gels. Bands were visualized under ultraviolet light after being stained with ethidium bromide and photographed.

CTX-M PCR product of 1,041 bp obtained with primers (CTXM3G F/CTXM3G R) (36) was sequenced as previously described and the nucleotide sequences and deduced-protein sequences were analyzed with the BLAST and FASTA programs of the National Center for Biotechnology Information (http://www.ncbi.nlm.nhi.gov).

Detection of insertion sequence IS*Ecp1B* was performed by PCR using primers IS*Ecp1*A and IS*Ecp1*B, which generate a 527 bp internal fragment of the transposase gene (38). Cycling conditions were as follows: initial denaturation at 94°C for 5 min followed by 30 cycles of denaturation at 94°C for 30 s, annealing at 58°C for 30 s and elongation at 72°C for 30 s. The final elongation step was extended to 10 min at 72°C.

The combination of IS*Ecp1B* primer (PROM+) with consensus primer CTX-MB (PROM+/CTXMB) was used to screen for genetic linkage between ISE*cp1B* and *bla*_CTX-M_ gene (38). PCR conditions were as follows: one cycle of 94°C for 5 min, 35 cycles of 94°C for 45 s, 56°C for 30 s, 72°C for 2 min and one final cycle of 72°C for 10 min.

*E. coli* TN03 carrying the IS*Ecp1*-like element upstream *bla*_CTX-M-15_ gene was used as a control.

### Conjugation experiment and plasmid analysis

Mating experiments were performed as previously described (3), with *E. coli* BM21 (NA resistant) as a recipient. Exponential cultures of ESBL positive isolates as the donor (1 vol) and recipient (2 vol) were inoculated as a spot on Brain Heart Infusion Agar (BHIA). After overnight incubation at 37°C, the bacteria were resuspended, diluted and plated onto BHIA containing relevant selective agents at the following concentrations: NA (50 μg mL^−1^) and CTX (4 μg mL^−1^). Samples from the donor and recipient were used as controls. Transconjugants growing on the selection plates were subjected to antibiotic susceptibility, DDST and PCR analysis.

Plasmid DNA was extracted by the alkalin lysis method as previously described (19) and analyzed by electrophoresis on 0.7% (w/v) agarose gels at 5 volts cm^−1^. Plasmid size was estimated by using reference plasmids from *E. coli* strain V517 (54, 5.6, 5.1, 3.9, 3, 2.7 and 2.1 kbp) and pRK2013 (48 kbp).

The incompatibility group of the plasmid was determined by the PCR-based replicon typing method developed by Carattoli *et al.*(6).

## Results

The values of pH, salinity and temperature at the sampling sites are typical of Mediterranean seawater in the spring (Table 1). Microbial load, suspended matter (SM) and BOD showed that seawater at the mouth of Reghaia’s lake, Ain Chrob, Ain Tagourait and Anse de Kouâli are from good to acceptable quality.

**Fig. 1:**
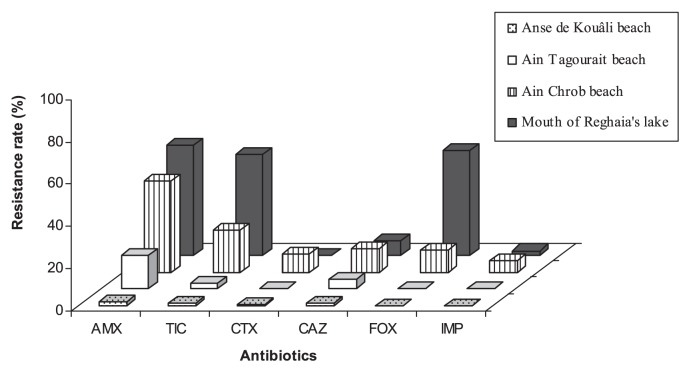
β-Lactam resistance among total flora from beach seawater. AMX: amoxicillin; TIC: ticarcillin; CTX: cefotaxime; CAZ: ceftazidime; FOX: cefoxitin; IMP: imipenem.

The evaluation of the prevalence of β-lactam resistance among total mesophilic flora showed the most significant rates for AMX and TIC, whereas the rates were low for CAZ, CTX and IMP. According to the sampling sites, the highest resistance rates were recorded for the sites subjected to chemical and microbiological inputs, namely the mouth of Reghaia’s lake (AMX: 52%, TIC: 47.7%, CTX: 2.7%, CAZ: 7.2%, FOX: 50%, and IMP: 2.2%) and Ain Chrob (AMX: 43%, TIC: 19.6%, CTX: 8.3%, CAZ: 11.3%, FOX: 10.3%, and IMP: 5.4%). For the sites relatively preserved from anthropogenic influence, resistance rates were lowest, it is the case for Ain Tagourait (AMX: 16%, TIC: 2.6%, CTX: 0.2%, CAZ: 4.5%, FOX: 0.1%, and IMP: 0.59%) and Anse de Kouâli (AMX: 1.5%, TIC: 0.8%, CTX: 0.6%, CAZ: 1%, FOX: 0.1%, and IMP: 0%) (Fig. 1).

Thirty-four bacteria isolated from samples from different beaches on medium supplemented with IMP (*n*=14) or CTX (*n*=20) were identified, tested for their resistance to 24 antibiotics, including 14 β-lactams, and analyzed for ESBL production. Their identification revealed the affiliation of IMP-resistant bacteria to the following species and genus (number of isolates): *Pseudomonas aeruginosa*(5), *Pseudomonas fluorescens*(2), *Stenotrophomonas maltophilia*(4), *Burkholderia cepacia*(2), *Pantoea* sp. (1) and those CTX-resistant to *P. aeruginosa*(10), *P. fluorescens*(5), *Bordetella* sp. (1), *Acinetobacter baumannii*(1), *Chryseomonas luteola*(1), *Ochrobactrum anthropi*(1) and *Escherichia coli*(1).

The activity of 24 antibiotics, including 14 β-lactams, against 14 IMP- and 20 CTX-resistant bacteria is shown in Table 2. Significant resistance rates were observed for AMX (100%), TIC (97%), FOX (97%), CFS (88.2%), CTX (94.1%), CRO (88.2%) and ATM (73.5%), followed by CAZ (50%), IMP (44.1%) and FEP (38.2%). A moderate resistance rate was observed for PIP (26.4%), mainly among IMP-resistant isolates. Almost all CTX-resistant isolates were susceptible to IMP. β-Lactamase inhibitors did not restore the activity of AMX, TIC and PIP significantly: AMC (76.4%), TCC (82.3%) and PTZ (20.5%).

Regarding non-β-lactams antibiotics, resistance rates were high for NA (85.2%) and TMP (82.3%), significant for C (55.8%) and TE (41.1%), moderate for SXT (14.7%), SSS (8.8%) and RA (17.6%), and low or zero for CIP (5.8%), K (2.9%) and GM (0.0%).

These tested isolates were characterized by 30 different multi-antibiotic resistance (MAR) profiles ranging from 9 to 20 antibiotics and covering 2 to 6 antibiotic families (Table 3).

Three isolates were positive for ESBL test, two IMP-resistant isolates of *S. maltophilia* from mouth of Reghaia’s lake and Ain Chrob, and one CTX-resistant *E. coli* isolate from Ain Chrob.

The mating assay performed on these isolates allowed the transference of ESBL phenotype from only the *E. coli* isolate to recipient *E. coli* BM21.

**Fig. 2:**
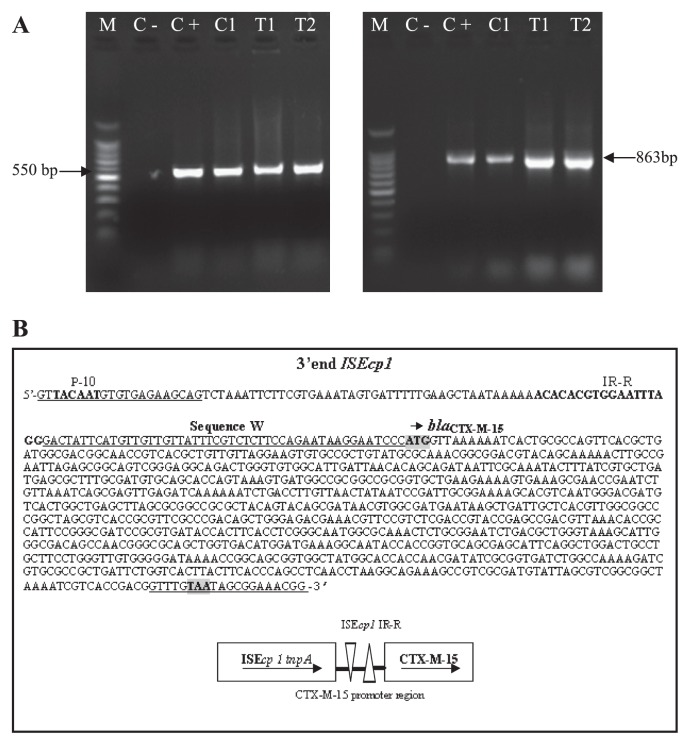
PCR products of *bla*_CTX-M_ gene (A) and map of the nucleotide sequence of *bla*_CTX-M-15_ gene (B) A: Detection of *bla*_CTX-M_ gene with consensus primers for family (left) and group 1 (right) in ESBL-positive *E. coli* (C1) and in its transconjugants (T1 and T2). The amplification band size of the *bla*_CTX-M_ family is 550 bp and for group 1 *bla*_CTX-M_ is 863 bp. C−: negative control; C+: positive control; M: 100 bp DNA marker. B: Map of the nucleotide sequence of the 3′ end of ISE*cp1* and *bla*_CTX-M-15_ and schematic representation of ISE*cp1*- *bla*_CTX-M-15_. The sequences at the 3′ and 5′ of the primers used for sequencing are underlined. P-10: sequence of CTX-M-15 promoter region, IR-R: right inverted repeat of ISE*cp1*, sequence W: intercalary sequence of 48 bp (underlined) between ISE*cp1* and *bla*_CTX-M-15_. Initiation and stop codons of *bla*_CTX-M-15_ are boxed in grey.

PCR amplification carried out on isolates ESBL+ and *E. coli* transconjugants showed the presence of ESBL belonging to the CTX-M-1 group in only *E. coli* and its transconjugants. The *bla*_TEM_ gene was not detected. Sequencing of CTX-M PCR-product revealed the presence of the CTX-M-15 allele (Fig. 2). An amplicon of 1,000 bp was obtained with primer combination CTX-M-B/PROM+, indicating the presence of the IS*Ecp1B* upstream CTX-M-15 gene and genetic linkage between them. lSE*cp1B*, whose transposase gene is oriented in the same direction as the *bla*_CTX-M-15_ gene, is separated from this one by an intercalary region of 48 bp corresponding to sequence W (Fig. 2).

**Fig. 3:**
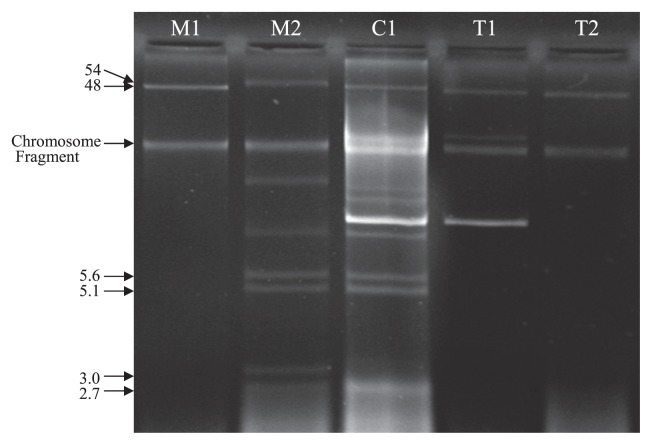
Plasmid profiles of ESBL-positive *E. coli* (C1) and its transconjugants (T1 and T2). M1: pRK2048 (48 kbp) and M2: *E. coli* V517 (54, 5.6, 5.1, 3.9, 3, 2.7 and 2.1 kbp)

Plasmid analysis showed that the CTX-M-15 gene is carried by the self-transferable plasmid of about 50 kbp belonging to incompatibility group IncI1 (Fig. 3).

## Discussion

The results showed that resistance rates were significantly higher on beaches receiving anthropogenic inputs (mouth of Reghaia’s lake and Ain Chrob) than on the unpolluted coast (Ain Tagourait and Anse de Kouâli). This corroborates the reports of Oliveira *et al.*(35), Mudryk *et al.*(32) and Manivasagan *et al.*(25) about the influence of human activities on the prevalence of bacterial antibiotic resistance in the marine environment. Indeed, antibiotic resistance is exacerbated by selective pressure and spreads through genetic exchange, under the influence of biotic and abiotic factors (9, 22, 25–27). High antibiotic resistance rates were noted in our study, particularly for AMX, TIC and FOX, and exceed those observed in clinical settings. It has been shown that environmental microbes (non-pathogenic or opportunistic pathogens) are often more antibiotic resistant than pathogens; therefore, their role as providers of resistance genes is under consideration (22, 47). Our results are in agreement with previous studies that have commonly reported β-lactam resistance in marine environments at high rates, especially for the penicillin group; resistance rates from 24.6% to 93.2% were reported for ampicillin (25, 27, 30, 31, 37), and a low incidence of resistance to IMP (16.5%) was noted (27).

In this study, we were interested in CTX- and IMP-resistant bacteria because of the importance of these antibiotics in human medicine and as markers of acquired β-lactam resistance mechanisms. Of the 34 IMP- or CTX-resistant isolates, except *E. coli* and *Pantoea* isolates, all other isolates were heterotrophic Gram-negative non-fermentative bacilli, indigenous to wide aquatic environmental sources; they are also known as opportunistic pathogens responsible for nosocomial infections (4, 24). In addition to resistance to IMP or CTX, these bacteria have marked resistance to most β-lactams (penicillins and second and third generation cephalosporins). Antibiotic resistance in aquatic environments is widely carried by Gram-negative rods (27). In a study of 16 rivers in USA, Ash *et al.*(1) found that ampicillin-, CTX-, CAZ- and IMP-resistant isolates were predominantly among Gram-negative nonlactose fermenters.

For non-β-lactam antibiotics, resistance rates were low to moderate for aminoglycosides, CIP, SSS, SXT and RA, and high for NA, TMP, C and TE. Tetracyclines are intensively used in agriculture and aquaculture to promote growth and in treatment and prophylaxis. Tetracycline resistance is well documented and is commonly described in seawater, mostly in Gram-negative bacteria (34, 42), at significant rates in terms of health risk (overall from 25% to 40%) around marine aquaculture sites (25, 34) and in coastal zones such as beaches exposed to terrestrial influences (25, 31, 37). High rates of resistance to NA and TMP have been reported (25, 27, 37), whereas chloramphenicol resistance varies across studies and a low incidence of resistance to aminoglycosides, RA and CIP, was noted (10, 16, 25, 29, 30, 31, 35, 37). As our strains were selected on medium supplemented with a β-lactam antibiotic, the high rates of resistance to TE, TMP and C observed may be the result of co-selection due to cross-resistance, as has been reported in several studies (8, 33).

Our isolates showed MAR profiles with 9 to 20 antibiotics; however, the number of antimicrobials to which strains were resistant was not necessarily related to the level of contamination of beaches, and this finding was not consistent, at least in part, with data reported by Oliveira *et al.*(35). MAR is found frequently in the marine environment (27, 30, 31, 35, 45), it is often plasmid mediated (45), and also can be the result of natural or acquired cross-resistance (14, 39).

For most of our isolates, MAR seemed to have an intrinsic nature, due to chromosomal cephalosporinase or penicillinase production combined with cell impermeability and efflux mechanisms (4, 39, 46). Indeed, the majority of our isolates (*n*=22) belonged to the genus *Pseudomonas*, which is characterized by its natural resistance to many β-lactams, including third generation cephalosporins, and non-β-lactams (TE, C and NA), and by its susceptibility to ureidopenicillins, carboxypenicillins, aminoglycosides and fluoroquinolones. *S. maltophilia* (*n*=4) produces metallo-β-lactamase L1 resistant to clavulanate and confers resistance to all β-lactams except ATM, and serine-β-lactamase L2, which is clavulanate susceptible and able to degrade penicillins, cephalosporins and ATM (2, 24).

The limited effect of β-lactamase inhibitors, particularly clavulanic acid, on resistance to penicillins means the non-production of clavulanate-sensitive β-lactamases, which is an acquired resistance mechanism encoded by plasmids (46).

MAR of our isolates can also result from genetic variation due to horizontal gene transfer by conjugation, transduction or transformation from contaminating bacteria of terrestrial influxes, and to efficient genetic recombination mediated by transposons and integrons. Ash *et al.*(1) and Glassman and McNicol (12) reported that over 40% of resistant bacteria from rivers and estuarine harbored plasmids, Biyela *et al.*(5) and Schmidt *et al.*(42) found class 1 integrons in over 50% and 67% of isolates from an urban river and fish farming environment respectively, and Rhodes *et al.*(40) described the implication of a transposon in the dissemination of tetracycline resistance in aquaculture environments. Resistance gene transfer in aquatic environments has been reported (9) and broad host range plasmids of IncP, IncQ and IncW groups play a leading role in enabling exchanges between phylogenetically distant bacteria (16, 47).

Our results are consistent with studies conducted in different aquatic environments that have concluded that drug resistance is emerging markedly in environmental bacteria in correlation with the human impact (22, 23). The characteristics of bacteria isolated on CTX- or IMP-supplemented media is their wide distribution in aquatic environments as saprophytes; however, they can be redoubtable opportunistic pathogens associated with nosocomial infections in debilitated patients, most commonly catheter-associated bacteremia (4). Their selection is due to their high natural resistance to antibiotics and also probably to their amplification by selective pressure exerted by abiotic factors (4, 22). The role of this environmental bacterial flora must be considered; indeed, under the influence of contaminants, they can accumulate genetic elements with clinical impact and may act as an amplifier and a reservoir of these elements (5, 43).

Screening for ESBL production using DDST showed a synergistic image between clavulanate and antibiotic markers (CTX, CRO, ATM and CAZ) for the CTX-resistant *E. coli* isolate and only with ATM for two IMP-resistant *S. maltophilia* isolates. This synergistic image is characteristic of chromosomic β-lactamase L2 for *S. maltophilia*(24), while it is indicative of ESBL production for *E. coli*. PCR analysis and nucleotide sequencing identified the ESBL of *E. coli* isolate as CTX-M-15. The epidemiology of ESBLs among clinical *Enterobacteriaceae* has recently changed with the massive dissemination of CTX-M-type enzymes, particularly CTX-M-15 allele; they are now the most widely distributed β-lactamases worldwide (15), with a well-established endemicity in some countries such as Algeria, where they predominate in clinical settings (28). To our knowledge, no study has described ESBLs in the marine environment, while in the terrestrial aquatic environment, CTX-M-14, CTX-M-15-like, CTX-M-1, CTX-M-4, CTX-M-27, and CTX-M-32 ESBLs have been reported (11, 21, 44).

ISE*cp*-1 insertion sequence was found upstream of the CTX-M-15 gene. This genetic organization has already been described in clinical isolates in which ISE*cp1B* is implicated in transposition and expression promotion of ESBL genes (38).

Mating experiments and plasmid analysis showed that the gene encoding CTX-M-15 was carried by a self-transferable plasmid of about 50 kbp of the IncI1 group. It was reported that CTX-M-15 has commonly been located on largely diffused Inc I1 plasmids whose occurrence is linked to selection exerted by antimicrobial use (17).

This work is the first description of antibiotic resistance in seawater on the beaches of Algeria, showing a significant level of resistance to antibiotics, particularly β-lactams, detected mainly among saprophytic environmental bacteria. This antibiotic resistance seems dependent on the level of anthropogenic inputs, which also affect the structure and composition of bacterial populations. Transmissible ESBL of CTX-M-15 type was detected in *E. coli*; this may mean that contamination of the environment by resistant bacteria may cause the spread of resistance genes, with the health risk from recreational water contact.

## Figures and Tables

**Table 1:** Physico-chemical and microbiological parameters of seawater samples

Parameters	Seawater sampling sites			

	Mouth of Reghaia lake	Ain Chrob	Ain Tagourait	Anse de Kouâli
Salinity (PSU)	32.2	36.8	36.8	36.7
pH	8.1	8.3	8.3	8.5
Temperature (°C)	13.6	14.3	14.5	14.3
Dissolved oxygen (mg L^−1^)	7.1	7.2	8.3	10.6
Oxygen saturation (%)	(76%)	(76%)	(81%)	(104%)
Suspended matter (mg L^−1^)	157.9	216.1	174.4	153.6
Biological oxygen demand (mg L^−1^)	5	5	0	0
Total flora (CFU 100 mL^−1^)	18,000	2,040	860	3,510
Total coliform (CFU 100 mL^−1^)	8,700	240	37	44
Thermotolerant coliform (CFU 100 mL^−1^)	400	1	25	19

**Table 2:** Antibiotic resistance rates among imipenem- or cefotaxime-resistant isolates

Resistant Isolates	Antibiotics																							

	β-lactams														Non β-lactams antibiotics									
	
	AMX	AMC	TIC	TCC	PIP	PTZ	CFS	FOX	CRO	CTX	CAZ	FEP	ATM	IMP	K	GM	SSS	TMP	SXT	TE	CIP	NA	C	RA
Imipenem (*n*=14)	14	7	13	10	7	6	13	14	10	12	6	7	8	14	0	0	1	13	1	10	2	12	10	6
Cefotaxime (*n*=20)	20	19	20	18	2	1	17	19	20	20	11	6	17	1	1	0	2	15	4	4	0	17	9	0
Total (*n*=34)	34	26	33	28	9	7	30	33	30	32	17	13	25	15	1	0	3	28	5	14	2	29	19	6
Resistance rate (%)	100	76.4	97	82.3	26.4	20.5	88.2	97	88.2	94.1	50	38.2	73.5	44.1	2.9	0	8.8	82.3	14.7	41.1	5.8	85.2	55.8	17.6

AMX, amoxicillin; AMC, amoxicillin/clavulanate; TIC, ticarcillin; TCC, ticarcillin/clavulanate; PIP, piperacillin; PTZ, piperacillin/tazobactam; CFS, cefsulodine; FOX, cefoxitin; CRO, ceftriaxone; CTX, cefotaxime; CAZ, ceftazidime; FEP, cefepim; ATM, aztreonam; IMP, imipenem; K, kanamycin; GM, gentamycin; SSS, sulfonamide; TMP, trimethoprim; SXT, trimethoprim/sulfamethoxazole; TE, tetracycline; CIP, ciprofloxacin; NA, nalidixic acid; C, chloramphenicol; RA, rifampicin.

**Table 3:** Antibiotic resistance traits of cefotaxime- and imipenem-resistant isolates

Imipenem- or cefotaxime-esistant isolates	Isolate	Origin	Resistance traits	Number of resistance traits	ESBL test
Imipenem-resistant isolates	I1: *P. fluorescens*	Ain Tagourait	AMX-TIC-TCC-CFS-FOX-IMP-TMP-NA-RA	09	−
	I2: *P. fluorescens*	Ain Chrob	AMX-AMC-FOX-CTX-IMP-TMP-SXT-TE-CIP-NA-C	11	−
	I3: *B. cepacia*	Ain Tagourait	AMX-TIC-TCC-CFS-FOX-CRO-CTX-ATM-IMP-TMP-C	11	−
	I4: *P. aeruginosa*	Mouth of Reghaia lake	AMX-TIC-TCC-CFS-FOX-IMP-TMP-TE-NA-C-RA	11	−
	I5: *P. aeruginosa*	Ain Chrob	AMX-TIC-TCC-CFS-FOX-CTX-IMP-TMP-TE-NA-C-RA	12	−
	I6: *P. aeruginosa*	Ain Chrob	AMX-TIC-TCC-CFS-FOX-CRO-CTX-IMP-TMP-TE-NA-C-RA	13	−
	I7: *P. aeruginosa*	Ain Chrob	AMX-AMC-TIC-PIP-PTZ-CFS-FOX-CRO-CTX-FEP-ATM-IMP-NA	13	−
	I8: *S. maltophilia*				+
	I9: *S. maltophilia*	Mouth of Reghaia lake	AMX-AMC-TIC-TCC-PIP-PTZ-CFS-FOX-CRO-CTX-CAZ-FEP-ATM-IMP-TMP	15	+
	I10: *B. cepacia*	Ain Chrob	AMX-AMC-TIC-PIP-TZP-CFS-FOX-CRO-CTX-CAZ-FEP-ATM-IMP-TMP-TE-NA	16	−
	I11: *S. maltophilia*	Ain Chrob	AMX-AMC-TIC-PIP-PTZ-CFS-FOX-CRO-CTX-CAZ-FEP-ATM-IMP-TMP-TE-NA-C	17	−
	I12: *S. maltophilia*	Ain Chrob	AMX-AMC-TIC-TCC-PIP-PTZ-CFS-FOX-CRO-CTX-CAZ-ATM- FEP-IMP-TMP-TE-NA-C	18	−
	I13: *P. aeruginosa*	Ain Tagourait			−
	I14: *Pantoea spp*	Ain Tagourait	AMX-TIC-TCC-PIP-PTZ-CFS-FOX-CRO-CTX-CAZ-FEP-ATM-IMP-SSS-TMP-TE-CIP-NA-C-RA	20	−

Cefotaxime-resistant isolates	C1: *E. coli*	Ain Chrob	AMX-TIC-CRO-CTX-SSS-TMP-SXT-C	8	+
	C2: *P. fluorescens*	Ain Chrob	AMX-AMC-TIC-TCC-CFS-FOX-CRO-CTX-ATM-NA	10	−
	C3: *Bordetella.* sp.	Ain Chrob	AMX-AMC-TIC-TCC-CFS-FOX-CRO-CTX-ATM-TMP-NA	11	−
	C4: *P. fluorescens*	Mouth of Reghaia lake	AMX-AMC-TIC-TCC-CFS-FOX-CRO-CTX-CAZ-ATM-NA	11	−
	C5: *P. fluorescens*	Mouth of Reghaia lake	AMX-AMC-TIC-TCC-CFS-FOX-CRO-CTX-CAZ-ATM-C	11	−
	C6: *P. aeruginosa*	Mouth of Reghaia lake	AMX-AMC-TIC-TCC-FOX-CRO-CTX-ATM-TMP-NA-C	11	−
	C7: *P. aeruginosa*	Ain Chrob	AMX-AMC-TIC-TCC-CFS-FOX-CRO-CTX-FEP-ATM-NA	11	−
	C8: *C. luteola*	Mouth of Reghaia lake	AMX-AMC-TIC-TCC-CFS-FOX-CRO-CTX-ATM-TMP-NA	11	−
	C9: *P. aeruginosa*	Mouth of Reghaia lake	AMX-AMC-TIC-TCC-CFS-FOX-CRO-CTX-CAZ-ATM-TMP-NA	12	−
	C10: *P. aeruginosa*	Mouth of Reghaia lake			−
	C11: *P. aeruginosa*	Ain Chrob	AMX-AMC-TIC-TCC-CFS-FOX-CRO-CTX-ATM-IMP-TMP-NA	12	−
	C12: *P. aeruginosa*	Mouth of Reghaia lake	AMX-AMC-TIC-TCC-CFS-FOX-CRO-CTX-ATM-TMP-NA-C	12	−
	C13: *P. fluorescens*	Mouth of Reghaia lake	AMX-AMC-TIC-TCC-CFS-FOX-CRO-CTX-CAZ-FEP-TMP-NA	12	−
	C14: *P. aeruginosa*	Mouth of Reghaia lake			−
	C15: *P. aeruginosa*	Mouth of Reghaia lake	AMX-AMC-TIC-TCC-CFS-FOX-CRO-CTX-CAZ-ATM-TMP-NA-C	13	−
	C16: *P. aeruginosa*	Mouth of Reghaia lake	AMX-AMC-TIC-TCC-CFS-FOX-CRO-CTX-ATM-TMP-TE-NA-C	13	−
	C17: *P. fluorescens*	Mouth of Reghaia lake	AMX-AMC-TIC-TCC-PIP-FOX-CRO-CTX-CAZ-ATM-TMP-SXT-TE-NA	14	−
	C18: *O. anthropi*	Ain Chrob	AMX-AMC-TIC-TCC-PIP-PTZ-CFS-FOX-CRO-CTX-CAZ-FEP- ATM-TMP-C	15	−
	C19: *P. aeruginosa*	Mouth of Reghaia lake	AMX-AMC-TIC-TCC-CFS-FOX-CRO-CTX-CAZ-FEP-ATM-TMP-SXT-TE-NA-C	16	−
	C20: *A. baumanii*	Ain Chrob	AMX-AMC-TIC-CFS-FOX-CRO-CTX-CAZ-FEP-ATM-K-SSS-TMP-SXT-TE-NA-C	17	−

AMX, amoxicillin; AMC, amoxicillin/clavulanate; TIC, ticarcillin; TCC, ticarcillin/clavulanate; PIP, piperacillin; PTZ, piperacillin/tazobactam; CFS, cefsulodine; FOX, cefoxitin; CRO, ceftriaxone; CTX, cefotaxime; CAZ, ceftazidime; FEP, cefepim; ATM, aztreonam; IMP, imipenem; K, kanamycin; GM, gentamycin; SSS, sulfonamide; TMP, trimethoprim; SXT, Trimethoprim/Sulfamethoxazole; TE, tetracycline; CIP, ciprofloxacin; NA, nalidixic acid; C, chloramphenicol; RA, rifampicin.
